# Spatiotemporal expression patterns of cytosolic AtHSP90-2 in *Arabidopsis* seedlings

**DOI:** 10.1080/15592324.2023.2202977

**Published:** 2023-04-18

**Authors:** Liudmyla Kozeko, Elizabeth Kordyum

**Affiliations:** Department of Cell Biology and Anatomy, M.G. Kholodny Institute of Botany of the National Academy of Sciences of Ukraine, Kyiv, Ukraine

**Keywords:** *Arabidopsis*, AtHSP90-2, gene expression pattern, developmental regulation, stress

## Abstract

Heat shock protein AtHSP90–2 is one of the three constitutive cytosolic HSP90s of *Arabidopsis thaliana*, which are highly homologous and show mild expression activation in response to stressful impacts. To characterize the functioning of *AtHSP90–2*, we have analyzed tissue-specificity of its expression during seedling development using a DsG transgenic line carrying a loss-of-function mutation of *AtHSP90–2* via translational fusions with the β-glucuronidase reporter gene (GUS). Histochemical analysis during the first two weeks of seedling growth revealed *AtHSP90–2* expression in all organs, as well as differences in its intensity between tissues and showed its dynamics. The tissue-specific *AtHSP90–2-GUS* expression pattern was shown to be maintained under heat shock and water deficit. The most prominent GUS staining was detected in the vascular system and hydathodes of cotyledons, and stipules. The basipetal gradient of *AtHSP90–2* expression during leaf formation, its dynamics in developing stipules, and the high level of its expression in cells with active transport function suggest a special role for the gene in certain cellular processes.

## Introduction

The heat shock proteins 90 kDa (HSP90) are a widespread, highly conserved family of molecular chaperones, which is essential in eukaryotes. They represent 1–2% of the cellular proteins under physiological conditions, and their amount increases in response to stressful impacts^1^. HSP90s mediate the maturation and functioning of a plethora of metastable proteins^2^. In plants, the set of HSP90-dependent proteins includes key components of diverse signal transduction pathways, cell cycle regulation, cell differentiation, etc.^3,4^.

Plant HSP90s are a multigene family with members of different subcellular compartments: cytosol/nuclear, plastid, mitochondria, and endoplasmic reticulum^5–8^. In *Arabidopsis thaliana*, the HSP90 family consists of seven isoforms, four of which are referred as cytosolic members: stress-inducible AtHSP90–1 (At5g52640), and constitutively expressed AtHSP90–2 (At5g56030), AtHSP90–3 (At5g56010) and AtHSP90–4 (At5g56000). Taking into account the high sequence similarity of cytosolic HSP90 (88–97%) in *A. thaliana*,^5^, their functional specificity and correspondent regulatory mechanisms have been considered. It has been shown that the various cytosolic HSP90s differ in their responsiveness to distinct stressors and show diverse expression kinetics^9–11^. Moreover, their expression is organ-specific and changes during plant development^9,12–15^. In particular, *AtHSP90–1* expression was determined in pollen grains and developing embryos under normal conditions and was strongly induced in all tissues under heat stress^12,13^. On the other hand, the three constitutive cytosolic members - *AtHSP90–2*, -*3* and *-4* are highly homologous and showed mild expression activation in response to stressful impacts^10,11^. A brief annotation of AtHSP90–2 and AtHSP90–3 on the Arabidopsis informational portal Araport (https://araport.org) describes their constitutive expression in all tissues and in abundance in root apical meristem, pollen, and tapetum^9^. Tight regulation of *AtHSP90–2* activity was demonstrated during embryogenesis and seed germination^13^At5g56030.1 was described as *AtHSP90–3*;^16^. The dynamics of activation of this gene in certain tissues was shown in detail in heat-shocked seedlings. It was first detected in the root tips of germinated seeds, and then appeared in the vascular system of 5-day-old seedlings and in the shoot meristem and cotyledon veins of 7-day-old seedlings^13^. However, we did not have much information about the dynamics of *AtHSP90–2* expression pattern under normal conditions. In this study, we focused on the spatial and temporal expression pattern of *AtHSP90–2* in *A. thaliana* during early development under normal and stressful (heat shock and water deficit) conditions.

## Materials and methods

Seeds of *Arabidopsis thaliana* (L.) Heynh. ecotype Columbia (Col-0) and the transgenic line GT_3_103910 (SM line, The JIC Gene Trap collection from the Exon Trapping Insert Consortium (EXOTIC) program) were obtained from the Nottingham Arabidopsis Stock Centre (NASC). GT_3_103910 provides a loss-of-function mutation of *AtHSP90–2* via translational fusions with the β-glucuronidase reporter gene (GUS). The Ds transposon is inserted within exon: 5 in At5g56030.1 and exon: 4 in At5g56030.2, 32 bp upstream of the 3’-UTR, in forward orientation (TAIR database). The insertion was preliminary verified according to the JIC protocol (http://signal.salk.edu/database/T-DNA/SM.435.pdf). Homozygous plants were revealed by PCR using the transposon-specific primer Spm32c (5’–TACGAATAAGAGCGTCCATTTTAGAGTGA-3’) and insertion site-specific primers (SMF, gene-specific primer: 5’–ACTTGCACTAACGCCAAGTTC–3’; SMR, specific to non-coding sequence: 5’–TGTCTTGTAACCGGCGAATAC–3’).

Surface-sterilized seeds were incubated at 4°C in the dark for 2 days and plated on the 0.8% agar substrate containing 0.5× MS medium and 1% (w/v) sucrose. Seedlings were grown in sterile conditions at 22 ± 1°C under long-day (16 h light/8 h dark) and light intensity 110 μmol m^−2^ s^−1^. Seedlings were analyzed after 3, 5, 8, 10 and 12 days of growth. For heat shock (HS), plates with 8- and 12-day-old seedlings were exposed to 37°C for 0.5, 2, 8, and 24 h. For water deficit, 6-day-old seedlings grown on medium I (1×MS, 4.5% sucrose, 1% agar) were transplanted onto medium II (0.25×MS, 1% agar) to avoid osmotic shock and subjected to progressive desiccation of the agar medium for 5 days as described earlier^17^.

RNA extraction and RT-PCR analysis of *AtHSP90–2* expression were carried out in Col-0 seedlings as described earlier^17^. Since *AtHSP90–1*, *-2*, *-3*, and *-4* are highly homologous, one pair of primers to their conserved sequences was used for PCR: F: 5’–GCTTTCCAAGCTGAGAT–3’, R: 5’–ACTTCCTCCATCTTGCT–3.’ To assess gene expression of *AtHSP90–2*, digesting of amplified cDNA for four genes was performed accordingly^18^. To assess *AtHSP90–2* mRNA level, equal amounts of cDNA were digested with restriction enzymes XhoI (ThermoSci) according to the manufacturer’s protocol. As a control, fragment of *AtUBQ5* (At3g62250) was amplified with primers: F: 5’–AACC-CTTGAGGTTGAATCATCC–3’, R: 5’–GTCCTTCTTTCTGGTAAACGT–3.’ Analysis was conducted in two independent biological samples with two analytical replicates.

For histochemical GUS staining, GT_3_103910 seedlings were fixed with 4% (v/v) formaldehyde in 0.1 M phosphate buffer, pH 7.0, for 30 min on ice, washed in the same buffer, and submerged in staining solution containing 1 mM ferricyanide, 1 mM ferrocyanide, 0.05% (w/v) Triton X-100, 0.05% (w/v) 5-bromo-4-chloro-3-indolyl-b-glucuronide cyclohexylamine in 0.1 M phosphate buffer, pH 7.0^19^ in modification of^20^. After vacuum infiltration, the staining reaction proceeded overnight at 37°C in the dark. Chlorophyll was removed by incubation in 70% ethanol, and then the tissue was rehydrated in water and mounted in 50% (v/v) glycerol for photography. The strain patterns were recorded using an Axio Vision Zeiss microscope (Germany) and a Canon 700D camera with a 100 mm Canon EF macro lens. Experiments with GUS staining were repeated three times with 8 to 20 plants examined per repetition.

## Results and discussion

**Figure 1.**
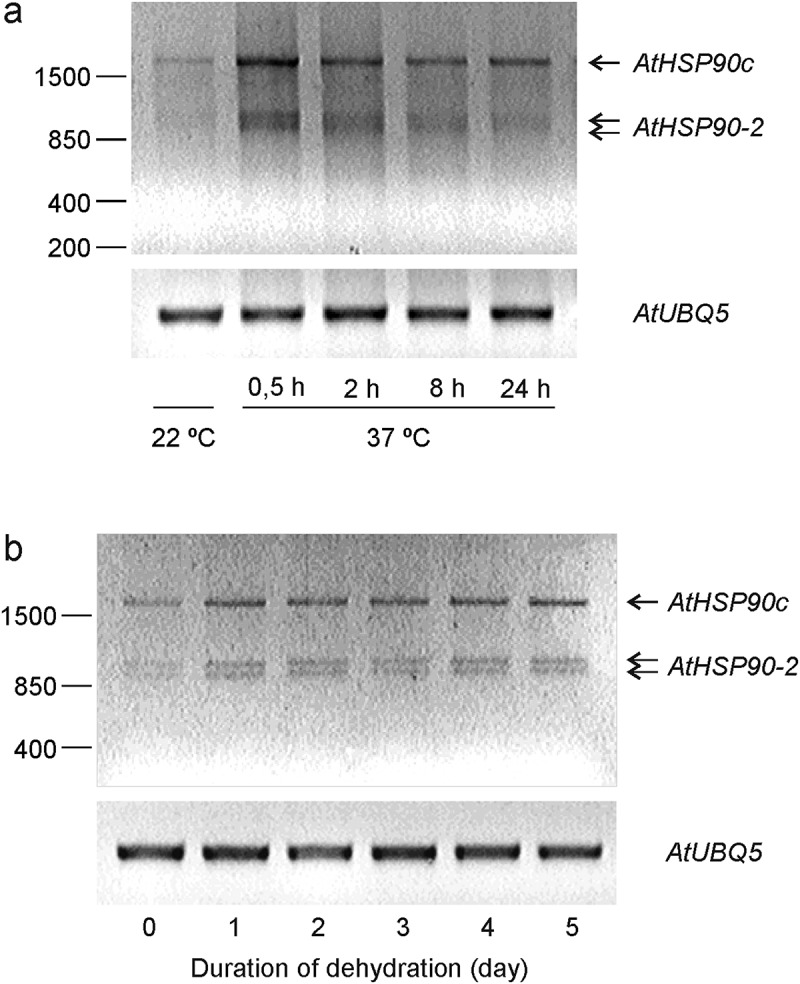
*AtHSP90–2* expression in Col-0 seedlings under heat shock (a) and dehydration (b). (a) 12-day-old seedlings were exposed to 37°C for different time periods (0.5–24 h). (b) Six-day-old seedlings were subjected to dehydration of the agar medium for 5 days. *AtHSPsp90c* indicates the RT-PCR amplification product of four cytosolic *HSPsp90s* (*AtHSP90–1*, -*2*, -*3* and -*4*). Equal amounts of the cDNA product were digested with restriction enzyme XhoI for *AtHSP90–2* (986 bp and 1086 bp are indicated with two arrows). *AtUBQ5* was used as an internal control. Representative images from two independent experiments are shown. (First published as a part of the Supplementary Information in Acta Physiologiae Plantarum, 2021, 43: 58 by Springer Nature).

Given the high homology of cytosolic *AtHSP90–1*, *-2*, *-3* and *-4* in *A. thaliana, AtHSP90–2* transcript level was determined by the restriction of amplified cDNA for the four HSP90s. The obtained results approved perceptible accumulation of *AtHSP90–2* transcript under normal conditions, as well as its moderate response to temperature increase^11,13,18^. The HS experiment showed that *AtHSP90–2* was induced at 37°C in a time-dependent manner, with a maximum after 30 min (Figure 1a). Transient up-regulation of GUS expression with a maximum after 3-h exposition at the same temperature was earlier shown by fluorometric analysis of GUS activity in *Arabidopsis* plants transformed with the promoter construct of this gene^13^. One of the sources for the differences in expression kinetics may be possible involvements of additional regulatory elements outside the promoter region used in the work cited above.

In the experiment with water deficit, *AtHSP90–2* expression was activated within the first day, and then remained at a fairly stable level for 5 days of dehydration of the growth medium (Figure 1b). In our opinion, the increase in its transcript level at the beginning of the experiment could likely be caused by the change of growth medium^17^. Then, the maintenance of a constant level of *AtHSP90–2* expression during the period of dehydration indicates that this gene is not involved in the response to water deficit, that is consistent with the absence of significant changes in its expression under sharp water shortage, osmotic stress, and ABA treatment^9,11^.

**Figure 2.**
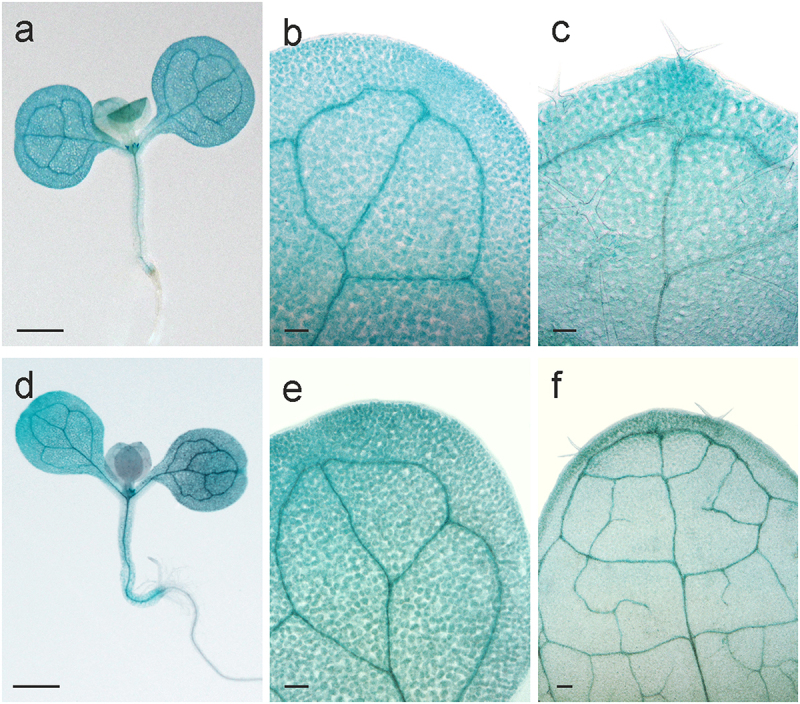
Expression patterns of *AtHSP90–2-GUS* in seedlings under (a-c) heat shock and (d-f) dehydration. (a-c) Histochemical detection of GUS activity in (a) 8-day-old seedling, (b and c) cotyledon and the first leaf of 12-day-old seedling, correspondingly, after exposition to 37°C for 2 h. (d-f) Histochemical detection of GUS activity in (a) 8-day-old seedling after 3 days of dehydration of the agar medium, (b and c) cotyledon and the first leaf of 11-day-old seedling, correspondingly, after 5 days of dehydration. Scale bars, 0.5 mm (a), 0.1 mm (b, c).

To visualize *AtHSP90–2* expression in the seedling tissue, we used the transgenic line GT_3_103910 with a transposon insertion in the third exon, 32 bp upstream of the 3’-UTR, which makes it possible to evaluate the expression of two transcript variants under the control of the chromosomal gene. In a heat-shock experiment, 8- and 12-day-old seedlings were exposed to 37°C for 2 h, giving a time lag for protein accumulation. Histochemical GUS staining revealed reporter gene activity in all organs, most prominent in cotyledons and leaves, especially in the vascular system and tips of the blades with hydathodes (Figure 2a–c), which is consistent with the results of Prasinos et al.^13^. A similar GUS expression pattern was observed in seedlings after dehydration for 5 days (Figure 2d–f).

**Figure 3.**
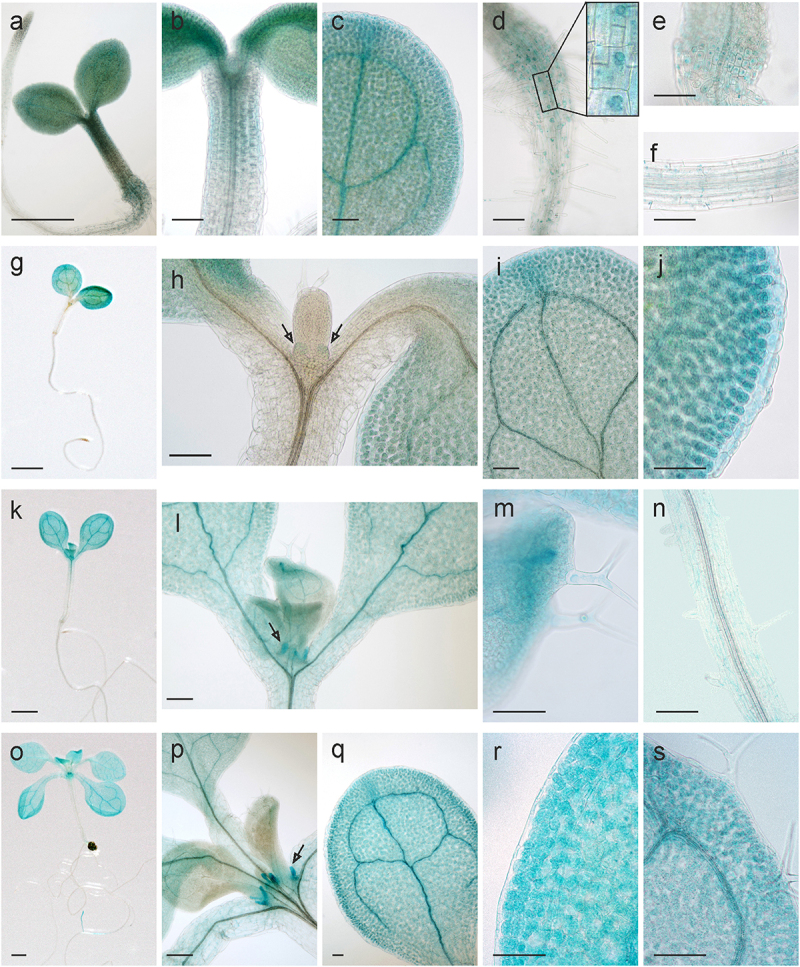
Expression patterns of *AtHSP90–2-GUS* during seedling development. Histochemical detection of GUS activity in 3-day-old (a – f), 5-day-old (g – j), 8-day-old ((k – n) and 12-day-old seedlings (o – s): (a, g, k, o) general view of seedlings, (b, h, l, p) shoot apex with developing stipules (pointed by arrows), (c, i, j, q, r) cotyledons, (m, s) the first leaf with trichomes, (d, e) root near its junction with the hypocotyl, (f, n) root. Scale bars, 1 mm (a, g, k, o), 0.1 mm (b-f, h-j, l-n, p-s).

Histological GUS analysis of seedlings grown under normal conditions was conducted at four growth stages, which reflect a range of morphological traits: 3-day-old seedlings – hypocotyl and cotyledon emergence (Figure 3a), 5-day-old seedlings – cotyledons opened fully (Figure 3g), 8-day-old seedlings – emergence of the first pair of leaves (Figure 3k), 12-day-old seedlings – the first pair of leaves formed fully (Figure 3o). Analysis of *AtHSP90–2-GUS* seedlings grown under normal conditions showed activity of the reporter gene in all organs. At the same time, differences in the intensity of its expression between tissues during seedling development were revealed. In 3-day-old seedlings, high levels of GUS staining were detected in the root, hypocotyl, mainly near its base and/or shoot apex, as well as in cotyledons, most prominent in the vascular system and hydathodes (Figure 3a–f). In 5-day-old seedlings, high level of *AtHSP90–2-GUS* expression was detected both in the epidermis and mesophyll of fully opened cotyledons (Figure 3i,j). Later, GUS activity remained strong in the mesophyll of cotyledons, but noticeably diminished in the epidermis (Figure 3r). In addition, weak staining was determined in stipules, formed beside the leaf primordia, while there was no obvious staining in the primordia themselves (Figure 3h). Later, the stipules at the base of the leaf reached bright blue color (Figure 3l,p). In 8-day-old seedlings, *AtHSP90–2-GUS* was expressed in cotyledons and stipules, as well as in the distal tip of not yet fully expanded leaves and trichomes on it, and weakly in the root (Figure 3k–n). In 12-day-old seedlings, strong GUS staining was detected in stipules, cotyledons, and leaves, most pronounced in the vascular system (Figure 3o–s). Such dynamics of *AtHSP90–2* expression pattern in the organs and tissues of seedlings indicates its spatiotemporal regulation at the early stages of plant development under normal conditions, as well as complements a similar pattern in heat-shocked *Arabidopsis* plants^13^.

It is noteworthy that the blue staining in the roots of 3-day-old seedlings was predominately localized in the nuclei (Figure 3e–f), but at the next terms, weak GUS activity was determined throughout the cytoplasm (Figure 3n). It is known that the amino acid sequences of cytosolic HSP90s of *A. thaliana* contain nuclear localization signal (NLS) and nuclear export sequence (NES)^21^. Earlier, prevailing nuclear localization was shown for *AtHSP90–1* and *AtHSP90–3* in root and hypocotyl cells of 7-day-old *A. thaliana* seedlings^21^. The presence and function of HSP90 in nucleus has been also described for animal cells^22,23^. The nuclear localization of cytosolic HSP90s are inhibited by geldanamycin^21^, i.e. it depends on ATP. Our observations indicate that the nuclear/cytosolic distribution of AtHSP90–2 may change during plant development.

**Figure 4.**
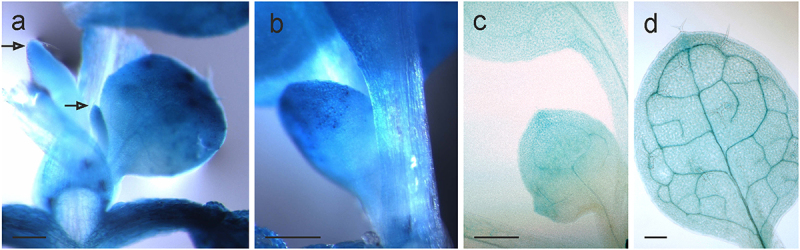
Expression patterns of *AtHSP90–2-GUS* in the first leaves. Histochemical detection of GUS activity (a) in leaf primordia (pointed by arrows), (b, c) expanding blade, (d) mature leaf.

It is remarkable that *AtHSP90–2-GUS* expression formed a longitudinal gradient in rosette leaves. GUS staining was first detectable in the tip of emerging leaves (Figure 4a), and gradually moved downward during leaf growth (Figure 4b,c), finally spreading throughout the leaf when it matured (Figure 4d). Interestingly, this gene expression dynamics resembles the basipetal gradient of the transition from cell proliferation to postmitotic cell expansion during *A. thaliana* leaf growth^24,25^. This transition is accompanied with great shifts in gene expression^25^. Accordingly, our data may reflect the participation of *AtHSP90–2* in maintaining the processes of expansion and differentiation of leaf cells. Supporting this assumption is the fact that HSP90 client proteins in plants include regulators of cell growth and differentiation^3,4^. In addition, stress-related genes were found to be up-regulated as the leaves matured, increasing their stress tolerance^26^.

Overall, our results demonstrated that *AtHSP90–2* expression was predominantly regulated in a tissue-specific manner during early development, which is maintained under heat shock and water deficit. Our results are consistent with the suggestion that the tissue specificity of the expression of constitutive cytosolic *HSP90s* are epistatic to heat-inducibility^9^, and reflect a correspondent hierarchy of regulatory mechanisms^13^. The basipetal gradient of its expression during leaf formation and its dynamics in stipules may suggest a distinctive role of the gene in functionally mature cells. Also, we can speculate that high levels of *AtHSP90–2* expression in the vascular system and hydathodes of leaves, as well as in the junction between the hypocotyl and root indicate a prominent role of the gene in cells with active transport function. These observations may indicate the involvement of *AtHSP90–2*, as well as protein encoded by it, in certain cellular processes, that needs further investigation of the regulatory mechanisms of tissue specificity of its expression and the search for target proteins for its function in certain tissues.

## References

[1] Lindquist S. Regulation of protein synthesis during heat shock. Nature. 1981;293:311–7. doi:10.1038/293311a0. PMID: 6792546. 6792546

[2] Zhao R, Davey M, Hsu YC, Kaplanek P, Tong A, Parsons AB, Krogan N, Cagney G, Mai D, Greenblatt J, et al. Navigating the chaperone network: an integrative map of physical and genetic interactions mediated by the Hsp90 chaperone. Cell. 2005;120(5):715–727. doi:10.1016/j.cell.2004.12.024. PMID: 15766533. 15766533

[3] Kozeko L. The role of HSP90 chaperones in stability and plasticity of ontogenesis of plants under normal and stressful conditions (*Arabidopsis thaliana*). Cytol Genet. 2019;53(2):143–161. doi:10.3103/S0095452719020063.

[4] Tichá T, Samakovli D, Kuchařová A, Vavrdová T, Šamaj J, Napier R. Multifaceted roles of HEAT SHOCK PROTEIN 90 molecular chaperones in plant development. J Exp Bot. 2020;71(14):3966–3985. PMID: 32293686. doi:10.1093/jxb/eraa177. 32293686

[5] Krishna P, Gloor G. The Hsp90 family of proteins in *Arabidopsis thaliana*. Cell Stress & Chaperon. 2001;6:238–246. doi:10.1379/1466-1268(2001)006<0238:thfopi>2.0.co;2. PMID: 11599565. PMC434405 11599565

[6] Xu J, Xue C, Xue D, Zhao J, Gai J, Guo N, Xing H. Overexpression of GmHsp90s, a heat shock protein 90 (Hsp90) gene family cloning from soybean, decrease damage of abiotic stresses in *Arabidopsis thaliana*. PLoS One. 2013;8(7):e69810. doi:10.1371/journal.pone.0069810. PMID: 23936107. 23936107 PMC3723656

[7] Chen J, Gao T, Wan S, Zhang Y, Yang J, Yu Y, Wang W. Genome-wide identification, classification and expression analysis of the HSP gene superfamily in tea plant (*Camellia sinensis*). Int J Mol Sci. 2018;19(9):2633. doi:10.3390/ijms19092633. PMID: 30189657. 30189657 PMC6164807

[8] Chaudhary R, Baranwal VK, Kumar R, Sircar D, Chauhan H. Genome-wide identification and expression analysis of Hsp70, Hsp90, and Hsp100 heat shock protein genes in barley under stress conditions and reproductive development. Funct Integr Genomics. 2019;19:1007–1022. doi:10.1007/s10142-019-00695-y. 31359217

[9] Yabe N, Takahashi T, Komeda Y. Analysis of tissue-specific expression of *Arabidopsis thaliana* Hsp90-family gene HSP81. Plant Cell Physiol. 1994;35:1207–1219. doi:10.1093/oxfordjournals.pcp.a078715. PMID: 7697294. 7697294

[10] Milioni D, Hatzopoulos P. Genomic organization of *hsp90* gene family in *Arabidopsis*. Plant Mol Biol. 1997;35:955–961. doi:10.1023/a:1005874521528. PMID: 9426614. 9426614

[11] Swindell WR, Huebner M, Weber AP. Transcriptional profiling of Arabidopsis heat shock proteins and transcription factors reveals extensive overlap between heat and non-heat stress response pathways. BMC Genomics. 2007;8:125. doi:10.1186/1471-2164-8-125. PMID: 17519032. 17519032 PMC1887538

[12] Haralampidis K, Miliony D, Rigas S, Hatzopoulos P. Combinatorial interaction of *cis* elements specifies the expression of the *Arabidopsis AtHsp90-1* gene. Plant Physiol. 2002;129:1138–1149. doi:10.1104/pp.004044. PMID: 12114568. 12114568 PMC166508

[13] Prasinos C, Krampis K, Samakovli D, Hatzopoulos P. Tight regulation of expression of two *Arabidopsis* cytosolic *Hsp90* genes during embryo development. J Exp Bot. 2005;56(412):633–644. doi:10.1093/jxb/eri035. 15582930

[14] Winter D, Vinegar B, Nahal H, Ammar R, Wilson GV, Provart NJ. An “electronic fluorescent pictograph” Browser for exploring and analyzing large-scale biological data sets. PLoS One. 2007;2(8):e718. doi:10.1371/journal.pone.0000718. PMID: 17684564. 17684564 PMC1934936

[15] Manitašević Jovanović S, Tucić B, Matić G. Differential expression of heat-shock proteins Hsp70 and Hsp90 in vegetative and reproductive tissues of *Iris pumila*. Acta Physiol Plant. 2011;33:233–240. doi:10.1007/s11738-010-0530-x.

[16] Samakovli D, Tichá T, Vavrdová T, Závorková N, Pecinka A, Ovečka M, Šamaj J. HEAT SHOCK PROTEIN 90 proteins and YODA regulate main body axis formation during early embryogenesis. Plant Physiol. 2021;186(3):1526–1544. doi:10.1093/plphys/kiab171. 33856486 PMC8260137

[17] Kozeko L. Different roles of inducible and constitutive HSP70 and HSP90 in tolerance of *Arabidopsis thaliana* to high temperature and water deficit. Acta Physiol Plant. 2021;43:58. doi:10.1007/s11738-021-03229-x.

[18] Yamada K, Fukao Y, Hayashi M, Fukazawa M, Suzuki I, Nishimura M. Cytosolic HSP90 regulates the heat shock response that is responsible for heat acclimation in *Arabidopsis thaliana*. J Biol Chem. 2007;282(52):37794–37804. doi:10.1074/jbc.M707168200. PMID: 17965410. 17965410

[19] Jefferson RA, Kavanagh TA, Bevan MW. GUS fusions: ß-glucuronidase as a sensitive and versatile gene fusion marker in higher plants. Embo J. 1987;6:3901–3907. doi:10.1002/j.1460-2075.1987.tb02730.x. PMID: 3327686. 3327686 PMC553867

[20] Vitha S, Beneš K, Phillips JP, Gartland KMA. Histochemical GUS Analysis. In: KMA G, and Davey M, editors. Agrobacterium Protocols. Methods in Molecular Biology. Vol. 44. Totowa, NJ: Springer; 1995. pp. 185–193. doi:10.1385/0-89603-302-3:185. 7581664

[21] Samakovli D, Margaritopoulou T, Prassinos C, Milioni D, Hatzopoulos P. Brassinosteroid nuclear signaling recruits HSP90 activity. New Phytol. 2014;203:743–757. doi:10.1111/nph.12843. PMID: 24807419. 24807419

[22] Gasc JM, Renoir JM, Faber LE, Delahaye F, Baulieu EE. Nuclear localization of two steroid receptor-associated proteins, HSP90 and p59. Exp Cell Res. 1990;186:362–367. doi:10.1016/0014-4827(90)90317-4. PMID: 2298246. 2298246

[23] Sawarkar R, Sievers C, Paro R. HSP90 globally targets paused RNA polymerase to regulate gene expression in response to environmental stimuli. Cell. 2012;149:807–818. doi:10.1016/j.cell.2012.02.061. PMID: 22579285. 22579285

[24] Donnelly PM, Bonetta D, Tsukaya H, Dengler RE, Dengler NG. Cell cycling and cell enlargement in developing leaves of *Arabidopsis*. Dev Biol. 1999;215:407–419. doi:10.1006/dbio.1999.9443. PMID: 10545247. 10545247

[25] Andriankaja M, Dhondt S, De Bodt S, Vanhaeren H, Coppens F, De Milde L, Mühlenbock P, Skirycz A, Gonzalez N, Beemster GT, et al. Exit from proliferation during leaf development in *Arabidopsis thaliana*: a not-so-gradual process. Dev Cell. 2012;22(1):64–78. PMID: 22227310. doi:10.1016/j.devcel.2011.11.011. 22227310

[26] Kanojia A, Gupta S, Benina M, Fernie AR, Mueller-Roeber B, Gechev T, Dijkwel PP, Foyer C. Developmentally controlled changes during Arabidopsis leaf development indicate causes for loss of stress tolerance with age. J Exp Bot. 2020;71(20):6340–6354. doi:10.1093/jxb/eraa347. 32720687 PMC7586751

